# The Experiences of Older Adults with Cannabis and Mental Health: A Scoping Review of the Literature

**DOI:** 10.1093/geroni/igab046.3533

**Published:** 2021-12-17

**Authors:** Raza Mirza, Amanda Bull, Andrea Gardiola, Sabina Mirza, Mary Hynes, Jessica Hsieh

**Affiliations:** 1 University of Toronto, Toronto, Ontario, Canada; 2 University of Toronto, Whitby, Ontario, Canada; 3 Queen's University, Toronto, Ontario, Canada; 4 York University, Woodbridge, Ontario, Canada

## Abstract

Following the 2018 federal legalization of cannabis in Canada, there was a drastic increase in older adults reporting marijuana use. Most cannabis research today focuses on children and young adults, however, it is important to acknowledge the potential harms in seniors as well. Aging and substance use presents unique considerations, such as the interactions between cannabis and chronic conditions, multiple comorbidities, polypharmacy, and mental health. The goal of this scoping review was to analyze the literature that addresses mental health outcomes of seniors who use cannabis, in order to answer the main research question: What is the relationship between older adults’ use of cannabis and mental health? Following Arksey and O’Malley’s five-stage framework, 10 electronic databases were searched along with a hand search of references. The search revealed 7000+ peer-reviewed and grey literature sources. 233 full-text sources were assessed for eligibility, with a total of 25 articles included. Thematic content analysis produced four major themes which addressed: (1) Usage characteristics; (2) User characteristics; (3) Outcomes; and (4) Physical and mental health considerations. Findings from this scoping review are positioned in terms of their implications for research, practice, and policy. While more in-depth, qualitative methods are required to develop further research, several harm-reduction strategies may be immediately utilized by both users and healthcare practitioners. It is critical that older adults and their physicians are able to make cannabis-related decisions with evidence-informed guidance to prevent problematic cannabis use and ensure positive mental health outcomes.

